# Health-related quality of life and self-related health in patients with type 2 diabetes: Effects of group-based rehabilitation versus individual counselling

**DOI:** 10.1186/1477-7525-9-110

**Published:** 2011-12-07

**Authors:** Eva S Vadstrup, Anne Frølich, Hans Perrild, Eva Borg, Michael Røder

**Affiliations:** 1 Department of Endocrinology and Gastroenterology, Bispebjerg University Hospital, Copenhagen, Denmark; 2 Department of Integrated Healthcare, Bispebjerg University Hospital, Copenhagen, Denmark; 3 Health Care Centre Østerbro, Copenhagen, Denmark; 4 Department of Cardiology and Endocrinology, Hillerød University Hospital, Hillerød, Denmark

## Abstract

**Background:**

Type 2 diabetes can seriously affect patients' health-related quality of life and their self-rated health. Most often, evaluation of diabetes interventions assess effects on glycemic control with little consideration of quality of life. The aim of the current study was to study the effectiveness of group-based rehabilitation versus individual counselling on health-related quality of life (HRQOL) and self-rated health in type 2 diabetes patients.

**Methods:**

We randomised 143 type 2 diabetes patients to either a six-month multidisciplinary group-based rehabilitation programme including patient education, supervised exercise and a cooking-course or a six-month individual counselling programme. HRQOL was measured by Medical Outcomes Study Short Form 36-item Health Survey (SF-36) and self-rated health was measured by Diabetes Symptom Checklist - Revised (DCS-R).

**Results:**

In both groups, the lowest estimated mean scores of the SF36 questionnaire at baseline were "vitality" and "general health". There were no significant differences in the change of any item between the two groups after the six-month intervention period. However, vitality-score increased 5.2 points (*p *= 0.12) within the rehabilitation group and 5.6 points (*p *= 0.03) points among individual counselling participants.

In both groups, the highest estimated mean scores of the DSC-R questionnaire at baseline were "Fatigue" and "Hyperglycaemia". Hyperglycaemic and hypoglycaemic distress decreased significantly after individual counselling than after group-based rehabilitation (difference -0.3 points, *p *= 0.04). No between-group differences occurred for any other items. However, fatigue distress decreased 0.40 points within the rehabilitation group (*p *= 0.01) and 0.34 points within the individual counselling group (*p *< 0.01). In the rehabilitation group cardiovascular distress decreased 0.25 points (*p *= 0.01).

**Conclusions:**

A group-based rehabilitation programme did not improve health-related quality of life and self-rated health more than an individual counselling programme. In fact, the individual group experienced a significant relief in hyper- and hypoglycaemic distress compared with the rehabilitation group.

However, the positive findings of several items in both groups indicate that lifestyle intervention is an important part of the management of type 2 diabetes patients.

## Background

Type 2 diabetes can seriously affect patients' health-related quality of life and their self-rated health. People with diabetes experience a decreased quality of life compared with people with no chronic illness but a better quality of life than people with most other serious chronic diseases [1]. The presence of two or more diabetes-related complications is associated with worsened quality of life [2] and lower scores of quality of life is associated with greater severity of complications for patients with type 2 diabetes [3]. Most often, evaluation of diabetes interventions assess effects on glycated haemoglobin (HbA_1c_) with little consideration of quality of life [4]. However, there is a growing interest in the assessment of health-related quality of life (HRQOL) in type 2 diabetes. An increasing number of type 2 diabetes trials, including studies evaluating diabetes self-management education, comprise measurements of quality of life [5]. The association between well-being and glycaemic control have been assessed in several studies. Some studies showed a positive effect on HRQOL in addition to improved glycaemic control [6-8] whereas others indicated a neutral or negative effect on HRQOL [9,10]. It is unknown whether impaired glycaemic control leads to lower quality of life or lower quality of life leads to impaired glycaemic control.

Group-based educational settings often encourage interaction and interpersonal dynamics and invite to social modelling compared to individual settings [11]. A small number of studies have compared the effects of group-based versus individual-based diabetes self-management programmes on HRQOL, but they found no significant differences between the groups [12,13]. Since quality of life is a multivariate phenomenon it has been suggested that evaluation should assess both generic and diabetes-specific elements of impairment including physical, emotional and social dimensions [14].

The Copenhagen Type 2 Diabetes Rehabilitation Project - a randomised controlled trial - was designed to study whether a six-month group-based rehabilitation programme improved glycaemic control in patients with type 2 diabetes compared with an individual counselling programme. The intervention used an empowerment-based approach and goal setting techniques [15]. Programme goals were to encourage behaviour changes, teach patients appropriate ways to exercise and improve nutrition, and strengthen patients' self-management skills. Previously, we demonstrated that both the rehabilitation programme and the individual counselling programme resulted in improved HbA_1c _levels, blood pressure and weight after the six months intervention period. However, HbA_1c _decreased significantly more after the individual counselling programme [16].

Secondarily we hypothesised that a group-based rehabilitation programme would result in a greater improvement in HRQOL and self-rated health than an individual counselling programme. The current paper evaluates the change in HRQOL and self-rated health after the six-month intervention period.

## Methods

### Study population

A detailed study design of The Copenhagen Type 2 Diabetes Rehabilitation Project have been published elsewhere [17]. Patients were recruited between August 2006 and February 2008 from our local outpatient clinic and general practitioners and by posting advertisements in local newspapers. Key inclusion criteria were: known or newly diagnosed type 2 diabetes, baseline HbA_1c _value between 6.8% and 10.0%, and ability to read and understand the Danish language. Key exclusion criteria were age less than 18 years, severe heart, liver or kidney disease, foot ulcers, and incurable cancer. Patients gave informed consent to participate in the study, which conformed to the principles of the Declaration of Helsinki, after which a baseline HbA_1c _was drawn. Patients fulfilling the inclusion criteria were randomised within three weeks stratified by gender and age. A person not participating in the study created a randomisation list. The investigator randomised and stratified the patients at the baseline visit using consecutively numbered sealed envelopes marked with gender (male or female) and age (< 55 years or > = 55 years). Patients were randomised to the group-based rehabilitation programme (rehabilitation group) at Healthcare Centre Østerbro or to the individual counselling programme (individual group) at the Diabetes Outpatient Clinic, Bispebjerg University Hospital. Neither patients nor study personnel were blinded to treatment assignment.

### Interventions

**The group-based rehabilitation programme**, conducted at a primary health care centre, was founded on evidence-based clinical guidelines [18] and emphasized a multidisciplinary approach. The programme used empowerment-based principles and goal-setting involving patient collaboration in order to improve the patients' knowledge and self-awareness [15]. Before patients entered the programme they participated in a motivational interview and set personal goals. Personnel were trained and supervised in the use of the motivational interviewing technique by an expert psychologist [19].

The programme consisted of an educational component of 90-minutes group sessions held weekly for a total of six weeks. Sessions were limited to eight patients and were taught by a nurse, a physiotherapist, a podiatrist, and a dietician. The educational curriculum included: the pathophysiology of diabetes, blood glucose self-monitoring, dietary instructions, the importance of physical activity, weight loss and smoking cessation, neuropathy, foot examinations, hypertension, complications, and medications [18]. A 12-week supervised exercise component consisted of 90-minutes sessions twice a week that included both aerobic and resistance exercise. The sessions were group-based, but a physiotherapist tailored an individual exercise programme for each patient. Dietary education included two three-hour group-based cooking classes and one two-hour session in a local supermarket.

The education, exercise, and dietary interventions could overlap and their sequence could differ from patient to patient. Goal achievement was evaluated in collaboration with the patients at the end of the intervention programme and one and three months after programme completion by telephone contacts.

**The individual counselling programme**, conducted at the diabetes outpatient clinic at Bispebjerg University Hospital, was based on the same clinical guidelines and the empowerment approach as in the primary health care centre [15,18]. The programme consisted of individual consultations with a diabetes nurse specialist, a dietician, and a podiatrist over a period of six months. All patients consulted the same nurse and dietician.

Patients participated in four one-hour sessions of individual counselling with a diabetes nurse specialist, who had a bachelor's degree in education and was trained in motivational interviewing [19]. Using the patients' own stories patients received personalized information and guidance about type 2 diabetes, medications, risk factors, and late complications, blood-glucoses self-monitoring, and increasing physical activity to the recommended level of 30 minutes of daily exercise. Over the same time period, patients participated in three individual counselling sessions with a dietician who was also trained in motivational interviewing [18]. At the initial hour-long visit, patients set personal goals and, in collaboration with the dietician, developed a dietary plan based on biochemical, anthropometrical and medical records and patients' motivation and attitudes. The action plan, progress towards meeting it, and goals were evaluated at the two follow-up visits, each of which lasted 30 minutes.

The endocrinologist or general practitioner caring for patients in both interventions prior to the study continued to provide diabetes management during and after the intervention; however, they were not part of the study team.

### Measurements

Patients filled in two self-administered questionnaires at baseline and at completion of the intervention. Patients were briefly provided with instructions on how to answer the questions.

The Medical Outcome Study 36-item Short Form Health Survey (SF-36 version 1.0) is a multi-purpose, short-form health survey with 36 questions that measure 8 conceptual domains: physical functioning, physical limitation, bodily pain, general health, vitality, social functioning, emotional limitation, and mental health [20]. The raw scores in each domain were transformed into 0 to 100 scales by the following calculation: (actual score - lowest possible score)/(possible score range) × 100. A higher score on SF-36 indicates better quality of life. The SF-36 has been proven useful in surveys of general and specific populations, comparing the relative burden of diseases, and in differentiating the health improvements produced by a wide range of different treatments [21]. The questionnaire has been translated into Danish and thoroughly validated in a Danish population [22].

As the SF-36 questionnaire is a generic measure, as opposed to one that targets a specific disease or treatment group, we included a diabetes specific questionnaire as a supplement. The Diabetes Symptom Checklist - Revised (DSC-R) is a self-report questionnaire measuring the occurrence and perceived burden of diabetes-related symptoms [23]. The DSC-R consists of 34 questions grouped into 8 symptom subscales: hyperglycaemia, hypoglycaemia, psychological cognitive functioning, psychological fatigue, cardiovascular symptoms, neuropathic pain, neuropathic sensory, and ophthalmologic functioning. Patients indicate whether they experienced any of the listed symptoms during the past month. For each symptom experienced, patients indicate the extent to which these symptoms were burdensome (ranging from "not at all", coded as 1, to "extremely", coded as 5). The eight subscale scores were calculated by summating the item scores, divided by the number of items of that subscale. A total symptom score was calculated from responses from all item score divided by 34. A lower score on DSC-R indicates less psychological and physiological distress. The DSC-R has been described to be valid, reliable and responsive to change and to be the only scale that appears to evaluate physical functioning in type 2 diabetes patients in a broad, comprehensive manner [24,25].

If patients skipped a question in the questionnaires the missing value was calculated as an average of rest of the values in the particular domain or subscale. A detailed description of the recorded demographic, laboratory, and clinical parameters has previously been published [16].

### Statistical analyses

The sample size calculation was based on the primary outcome (HbA_1c_) in the study. Using a target between-group absolute difference in HbA_1c _of 0.7%, a standard deviation of 1.3%, a power of 0.9, and a two-sided α of 0.05, we calculated a necessary sample size of 80 patients in each group. However, due to time and resources constraints, we were able to randomize 70 patients to the rehabilitation group and 73 patients to the individual group.

All available data were used in the analysis. Since 24 patients did not complete the baseline questionnaires it was not possible to include them in the intention-to-treat analysis. Hence, an intention-to-treat analysis was performed including patients lost to follow-up.

Differential changes between the two groups were analysed using a two-way analysis of variance with adjustment for baseline values in SAS, version 9.1 (Cary, NC). The study statistician performing the data analyses was blinded to patients' assignment to the rehabilitation group or individual group. Statistical significance level was set at *p  *< 0.05.

### Statement of ethics

The Danish National Committee on Biomedical Research Ethics and the Danish Data Protection Agency approved the study protocol. ClinicalTrials.gov registration number: NCT00284609.

## Results

**Figure 1:**
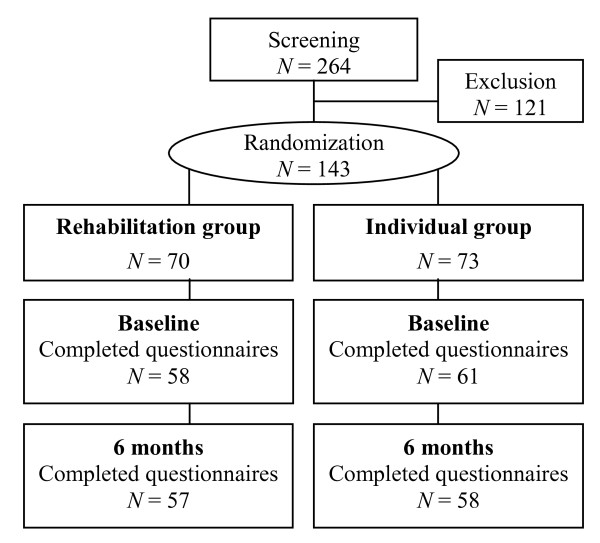
**The study flow shown for HRQOL (Health-related Quality of Life) and self-rated health assessments**. Of the 264 patients screened 121 was excluded mainly due to too low HbA_1c_. Main reasons for missing data after randomisation and during the intervention period were dropout due to time constraints and disappointment with randomisation and lost questionnaires.

Of 264 individuals who were screened, 143 met the inclusion criteria and were randomised. The vast majority of screen failures were due to HbA_1c _below 6.8%. Baseline characteristics of patients in the two groups were comparable (Table 1). Twenty-eight (20%) patients dropped out from the study (12 from the rehabilitation group and 16 from the individual group) of which six patients agreed to participate in the six-month follow-up visit. Reasons for dropping out were mainly due to time constraint and disappointment with the randomisation. The baseline characteristics of the patients who were missing or lost to follow-up did not differ significantly from the overall baseline characteristics of patients who completed the interventions, with the single exception that drop-outs in the individual group had higher weight (114.0 kg versus 95.0 kg, *p *< 0.05) and waist circumference (120.2 kg versus 106.1 kg, *p *< 0.05) than completers. The proportions of patients completing both questionnaires are shown in Figure 1.

**Table 1:** Descriptive characteristics of participants at baseline by group

	Rehabilitation group	Individual group
N	70	73
Male/Female	41/29 (59/41)	44/29 (60/40)
Age, years	58.5 ± 9.0	58.0 ± 10.3
Diabetes duration, years (range)	6.7 (0-37)	6.4 (0-24)
- Newly diagnosed diabetes	14 (20)	12 (15)
HbA_1c_, %	7.9 ± 0.8	7.8 ± 0.9
Weight, kg	96.2 ± 15.2	98.2 ± 24.8
Smokers/Ex-smokers	15/27 (21/39)	13/36 (18/49)
No antidiabetic drugs	9 (13)	17 (23)
OAD only	48 (68)	46 (63)
Insulin	13 (19)	10 (14)
Microalbuminuria	9 (13)	14 (19)
Macroalbuminuria	3 (4)	3 (4)
Retinopathy	4 (6)	3 (4)
Peripheral neuropathy	28 (40)	24 (33)
Cardiovascular event	8 (11)	9 (12)

Mean ± SD or N (%). OAD: Oral Antidiabetic Drug. Microalbuminuria was defined as a urine Albumin:Creatinine Ratio (ACR) ≥ 2.5 - 25 mg/mmol in men and ≥ 3.5 - 25 mg/mmol in women. Macroalbuminuria: ARC > 25 mg/mmol. Peripheral neuropathy was defined as biothesiometric value > 25 volt. Cardiovascular event: Myocardial infarction, Coronary revascularization, Angina pectoris or Stroke.

Adherence to the intervention programmes was judged by session attendance. In the rehabilitation group, 37 (64%) patients attended at least 18 of 24 exercise sessions, 42 (72%) patients attended at least five of six education sessions, and 50 (86%) patients attended at least two of three dietary education sessions. In the individual group, 48 (84%) patients attended at least three of the four nurse counselling sessions, and 50 (88%) patients attended at least two of the three dietician counselling sessions.

### SF-36

In both groups, the lowest estimated mean scores at baseline were "vitality" and "general health" (Table 2). There were no significant differences in the change of any item between the two groups after the 6-months intervention period. However, the mean score of vitality tended to increase within the rehabilitation group (by 5.2 points, *p *= 0.12) and increased significantly within the individual group (by 5.6 points, *p *= 0.03). In all other items the increases were small and did not reach a statistical significant level.

**Table 2:** SF-36 outcomes at baseline and after 6 months intervention.

	Rehabilitation group		Individual group			
			
	Baseline n = 58	6 months n = 57	Baseline n = 60	6 months n = 58	Model summary^‡ ^(95% CI)	*P*^§^
Physical function	78 (19)	83 (18)	83 (20)	87 (15)	1.0 (-4.1 to 6.1)	0.70
Limitation due to physical problems	72 (35)	78 (34)	73 (37)	78 (34)	-1.5 (-13.5 to 10.6)	0.81
Bodily pain	75 (26)	76 (26)	77 (23)	82 (22)	3.5 (-5.0 to 12.0)	0.42
General health	63 (21)	68 (19)	65 (17)	69 (17)	0.2 (-5.3 to 5.6)	0.96
Vitality	59 (24)	66 (24)	63 (23)	69 (20)^¶^	1.5 (-5.6 to 8.6)	0.68
Social functioning	87 (24)	85 (24)	89 (17)	89 (18)	2.4 (-5.2 to 10.0)	0.53
Limitation due to emotional problems	78 (33)	81 (29)	74 (39)	82 (34)	2.2 (-8.8 to 13.2)	0.69
Mental health	77 (19)	80 (18)	79 (18)	82 (16)	0.9 (-4.5 to 6.2)	0.74

Data are means (SD). Score scale range (0-100). A higher score indicates an improvement. ^‡^Difference in the change (from baseline to 6 months) of each variable *between *the two groups, when adjusted for baseline values. ^§^Significance of the difference between groups. ^¶ ^Significant (*P *< 0.05) difference from baseline to 6 months *within *the group.

### DSC-R

The estimated means of self-rated health from the DSC-R questionnaire at baseline and after the 6-months intervention period are shown in Table 3. In both groups, the highest estimated mean scores at baseline were "Fatigue" and "Hyperglycaemia". After the 6-months intervention period hyperglycaemic and hypoglycaemic distress were significantly improved in the individual group compared with the rehabilitation group (difference -0.3 points, *p *= 0.04). There were no differences between the two groups in any of the other symptom scales. However, in each group fatigue distress significantly improved (by -0.40 points, *p *= 0.01, in the rehabilitation group and by -0.34 points, *p *< 0.01, in the individual group). In the rehabilitation group cardiovascular distress significantly decreased by -0.25 points (*p *= 0.01). In the individual group hyperglycaemic distress significantly decreased by 0.31 points (*p *= 0.02) and hypoglycaemic distress significantly decreased by 0.28 points (*p *= 0.02).

**Table 3:** DSC-R outcomes at baseline and after 6 months intervention

	Rehabilitation group		Individual group			
			
	Baseline n = 58	6 months n = 57	Baseline n = 60	6 months n = 58	Model summary^‡ ^(95% CI)	*P*^§^
Hyperglycaemia	1.4 (1.3)	1.3 (1.1)	1.5 (1.1)	1.2 (1.1)^¶^	-0.33 (-0.65 to -0.02)	0.04
Hypoglycaemia	1.1 (1.1)	1.1 (1.1)	1.0 (1.0)	0.7 (0.8)^¶^	-0.30 (-0.60 to -0.01)	0.04
Fatigue	2.1 (1.3)	1.6 (1.2)^¶^	1.8 (1.2)	1.5 (1.1)^¶^	-0.02 (-0.37 to 0.32)	0.89
Cognitive	1.2 (1.1)	1.1 (1.1)	1.0 (0.9)	0.9 (0.8)	-0.12 (-0.36 to 0.12)	0.33
Pain	0.6 (0.9)	0.6 (1.0)	0.4 (0.8)	0.5 (1.0)	-0.05 (-0.41 to 0.30)	0.76
Sensory	0.7 (0.7)	0.6 (0.8)	0.5 (0.7)	0.5 (0.9)	0.09 (-0.17 to 0.35)	0.49
Cardiology	0.9 (0.9)	0.6 (0.8)^¶^	0.7 (0.7)	0.6 (0.7)	0.13 (-0.09 to 0.35)	0.23
Vision	0.6 (0.7)	0.6 (0.8)	0.5 (0.8)	0.5 (0.9)	-0.02 (-0.28 to 0.25)	0.90
Total	1.0 (0.6)	0.9 (0.7)	0.9 (0.6)	0.8 (0.6)	-0.04 (-0.21 to 0.13)	0.61

Data are means (SD). Score scale range (0-5). A lower score indicate an improvement. ^‡^Difference in the change (from baseline to 6 months) of each variable *between *the two groups, when adjusted for baseline values. ^§^Significance of the difference between groups. ^¶ ^Significant (*P *< 0.05) difference from baseline to 6 months *within *the group.

The change in hyperglycaemic distress was significantly correlated to change in HbA_1c _levels (Spearman rank-correlation coefficient of 0.29, *P *< 0.01) suggesting a lower frequency of hyperglycaemic symptoms and an improvement in hyperglycaemic distress with lower HbA_1c _levels.

### Intention-to-treat analysis

When the analysis was repeated as an intention-to-treat analysis the number of comparisons used only increased from 107 to 119 and all results on health-related quality of life and self-rated health remained unchanged.

## Discussion

A 6-months group-based rehabilitation programme did not improve HRQOL or self-related health in type 2 diabetes patients more than after individual counselling. In fact, the individual group experienced a significant relief in hyper- and hypoglycaemic distress compared with the rehabilitation group. Both groups reported less fatigue distress and increased vitality after six months compared with baseline.

At baseline, the most burdensome symptoms in our study population of type 2 diabetes patients were related to low vitality in the SF-36 questionnaire and fatigue in the DSC-R questionnaire. This was also found in studies evaluating the questionnaires in both type 2 diabetes patients [8,23,26,27] and in the general population [28]. However, the mean score of several items in the SF-36 questionnaire was lower in our study population compared with the general Danish population but higher compared with a study population of uncontrolled type 2 diabetes patients [8,28]. The mean score of several items in the DSC-R questionnaire was lower in our population compared with newly diagnosed type 2 diabetes patients but higher than a population of insufficient controlled type 2 diabetes on oral therapy [26,27].

The mean baseline score of the vitality scale (61 point) in the overall study population was lower than in the general Danish population (69 point) [28]. Although not statically significant, the mean score of vitality increased by approximately 5 point in both groups after the interventions. A study by Bjørner et al. interpreted score differences in the SF-36 vitality scale in patients with chronic conditions [29]. Patients suffering from a condition with a 5-point lower vitality score (compared with patients without that condition) had significantly increased odds of inability to work (odds ratio, OR, 1.27), job loss within 1 year (OR 1.13) and hospitalisation within 1 year (OR 1.08). Patients with diabetes had especially high OR for hospitalisation (OR 1.63). The improvements in the other SF-36 scales were between 0 and 4 points except for social functioning that deteriorated. A reasonable argument could be that a 6-months intervention period might not be enough time to improve social and emotional functioning. However, in the UK Prospective Diabetes Study there were no significant differences in the average changes of HRQOL over a six-year period between patients allocated to conventional versus intensive treatment [10]. The baseline SF-36 scores are relatively high, reflecting a patient population who has relatively good health and functional status. This in itself might explain the small improvement. Another explanation could be that it is more difficult to show differences in a generic questionnaire than in a disease specific questionnaire following education or self-management interventions [30]. Therefore it is important to use a questionnaire designed for the population of interest.

In the DSC-R questionnaire fatigue distress were improved within both groups after the interventions. The individual group reported significantly less hyperglycaemic and hypoglycaemic distress compared with baseline values and compared with the rehabilitation group. The magnitude of these improvements ranged from 0.28 to 0.40 points which is close to the minimal important difference ranged from 0.39 to 0.60 point estimated in a psychometric evaluation of the DSC-R questionnaire [24]. The rehabilitation group reported less cardiovascular distress after the intervention, which might be a result of the included exercise in the group-based rehabilitation programme.

We found an improvement in glycaemic control in both intervention groups [16]. As some studies showed a positive effect on HRQOL outcomes in addition to improved glycaemic control we had expected to find more significant improvements in HRQOL outcomes in our study [6-8]. In addition, a meta-analysis comparing didactic educational programmes with self-management educational programmes found that HRQOL improved more following self-management education [30]. Due to group interaction and interpersonal dynamics in the rehabilitation group we had expected larger improvements in HRQOL outcomes between the two groups in favour of the rehabilitation group. However, our results are consistent with other studies assessing the effect of group-based self-management education programmes on HRQOL founding no difference between intervention and control groups [12,13,31,32].

The study is limited by the high frequency of non-completers. Even at baseline 17% of the patients did not complete the questionnaires. The patients were asked to complete the questionnaires at home after the randomisation and then bring it back to the study personnel on the first day of the intervention. Most of the lost patients dropped out at the time of randomisation and refused to fill in the questionnaires and therefore we do not have any baseline values of these patients. Because the majority of results obtained in both groups were similar, any selection bias is likely to have been small. In addition, confidence intervals were generally wide (Table 2 and 3) and might indicate an inadequate sample size and a type 2 error. From the overall baseline characteristics we found that patients who were missing or lost to follow-up only had higher weight and waist circumference compared with completers. This suggests that no-response bias might not be an important factor influencing the results of the questionnaires. Limitations of our study also include the fact that it was not possible to identify the effect of each component of the interventions.

The present study was strengthened by the use of both a validated diabetes symptom questionnaire and a well-established generic quality of life questionnaire. We used a randomised controlled design to compare the effects on both clinical and self-reported outcomes of two lifestyle intervention programmes for type 2 diabetes patients. Our study can be regarded as a 'real life' trial much reflecting the clinical care setting and therefore the results are in line with what is possible to obtain in non-research settings.

## Conclusions

This study suggests that a group-based rehabilitation programme is not superior to an individual counselling programme in changing patients' HRQOL and self-rated health. This is interesting taking into account that the personnel resource use in the rehabilitation programme was twice as much as in the individual programme. However, the positive findings of several items in both groups indicate that lifestyle intervention is an important part of the management of type 2 diabetes patients. Long-term follow-up results of this study will determine whether or not the improvements are sustainable.

## Abbreviations

HbA_1c_: glycated haemoglobin; HRQOL: health-related quality of life; SF-36: Medical Outcomes Study Short Form 36-item Health Survey; DSC-R: Diabetes Symptom Checklist - Revised;

## Competing interests

The authors declare that they have no competing interests.

## Authors' contributions

ESV drafted the manuscript. All authors participated in the design of the study and provided input into the main ideas of this paper. All authors obtained funding for the project. ESV carried out screening, randomization and examination of the patients, and performed part of the statistical analysis. All authors read, commented, and approved the final version of the manuscript.
